# Does robot-assisted surgery reduce leg length discrepancy in total hip replacement? Robot-assisted posterior approach versus direct anterior approach and manual posterior approach: a propensity score-matching study

**DOI:** 10.1186/s13018-023-03864-9

**Published:** 2023-06-22

**Authors:** Mingyang Ma, Ping Song, Shuai Zhang, Xiangpeng Kong, Wei Chai

**Affiliations:** 1 Chinese PLA Medical School, Beijing, China; 2 Department of Orthopedics, The Fourth Medical Center of Chinese PLA General Hospital, Fuxing Road No. 28, Haidian, Beijing, China; 3 National Clinical Research Center for Orthopedics Sports Medicine and Rehabilitation, Beijing, China

**Keywords:** Total hip replacement, Leg length discrepancy, Propensity score-matching study

## Abstract

**Background:**

Advocates of robot-assisted technique argue that robots could improve leg length restoration in total hip replacement. However, there were few studies to compare the robot-assisted posterior approach (RPA) with conventional posterior approach (PA) THA and direct anterior approach (DAA) THA in LLD. This study aimed to determine whether robot-assisted techniques could significantly reduce LLD compared to manual DAA and manual PA.

**Methods:**

We retrospectively reviewed the cohort of consecutive ONFH patients who underwent THA robot-assisted posterior, manual posterior, and manual DAA from January 2018 to December 2020 in one institution. One experienced surgeon performed all procedures. We calculated the propensity score to match similar patients in different groups by multivariate logistic regression analysis for each patient. We included confounders consisting of age at the time of surgery, sex, body mass index (BMI), and preoperative LLD. Postoperative LLD and Harris hip scores (HHS) at two years after surgery of different cohorts were compared.

**Result:**

We analyzed 267 ONFH patients treated with RPA, DAA, or PA (73 RPA patients, 99 DAA patients, and 95 PA patients). After propensity score matching, we generated cohorts of 40 patients in DAA and RPA groups. And we found no significant difference in postoperative LLD between RPA and DAA cohorts (4.10 ± 3.50 mm vs 4.60 ± 4.14 mm, *p* = 0.577) in this study. The HHS at 2 years postoperatively were 87.04 ± 7.06 vs 85.33 ± 8.34 *p* = 0.202. After propensity score matching, we generated cohorts of 58 patients in manual PA and RPA groups. And there were significant differences in postoperative LLD between the RPA and PA cohorts. (3.98 ± 3.27 mm vs 5.38 ± 3.68 mm, *p* = 0.031). The HHS at 2 years postoperatively were 89.38 ± 6.81 vs 85.33 ± 8.81 *p* = 0.019. After propensity score matching, we generated cohorts of 75 patients in manual DAA and PA groups. And there were significant differences in postoperative LLD between the DAA and PA cohorts. (4.03 ± 3.93 mm vs 5.39 ± 3.83 mm, *p* = 0.031) The HHS at 2 years postoperatively were 89.71 ± 6.18 vs 86.91 ± 7.20 *p* = 0.012.

**Conclusion:**

This study found no significant difference in postoperative LLD between RPA and DAA, but we found a significant difference between RPA and manual PA, DAA and manual PA in ONFH patients. We found a significant advantage in leg length restoration in primary total hip arthroplasty with robot-assisted surgery.

## Introduction

Total hip arthroplasty (THA) is one of the most successful surgeries in modern medicine [1]. Hip replacement has revolutionized the treatment of advanced osteonecrosis of the femoral head (ONFH) with excellent outcomes. However, the leg length discrepancy (LLD) after THA has been associated with overall dissatisfaction [2–4] and was identified as the leading cause of litigation against orthopedic surgeons [5–7].

The posterior approach (PA) is mainstream in THA because it is safe, easy to perform, and highly reliable in complex cases. However, positioning the patient in a lateral position is challenging to assess the length of the limb. Furthermore, the PA takes more soft tissue release. Surgeons always sacrifice leg length equality for additional stability when we use the posterior approach. The direct anterior approach (DAA) THA has become increasingly popular because of its advantages in shorting hospital length of stay [8] and low dislocation rate [9, 10]. Placing the patient supine is advantageous in evaluating the range of motion and limb lengths [11–13].

To date, only a few studies have compared RPA with convention PA or DAA-THA in terms of LLD [14, 15]. However, their study had significant differences at baseline and lacked matching or had too small sample sizes. Their conclusions seem to be unreliable.

Therefore, we conducted this study to determine whether a robot-assisted technique significantly reduces LLD compared to DAA and manual posterior approach in matched primary THA cohorts. The hypothesis of this study was that RPA might provide a better LLD than PA, similar to DAA.

## Patients and methods

Figure 1.

**Fig. 1:**
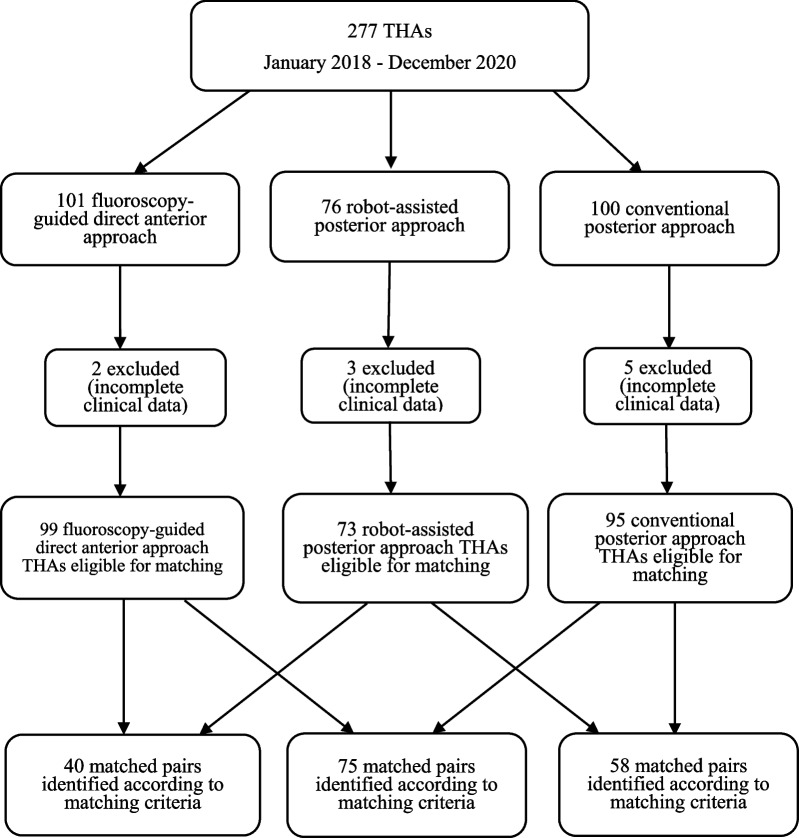
The flowchart shows the total number of THAs performed during the study period and the numbers of THAs performed using each technique

### Inclusion and exclusion criteria

Institutional Review Board approval of the study was obtained. The consecutive cases that underwent THA through RPA, DAA, and manual PA were reviewed from January 2018 to December 2020. All data were obtained from medical records. Study inclusion criteria were (1) patients with diagnosis of ONFH and (2) operation performed by one surgeon, (3) the patient have availability of postoperative pelvis radiographs and complete medical records, and (4) the surgery was operated with the robot-assisted posterior approach, DAA, or manual PA. Exclusion criteria were (1) incomplete clinical data or missing proper postoperative radiographs [16] (radiographs with rotated or tilted pelvis, the included angle between the axial line of femoral marrow cavity and the median line was more extensive than 10 degrees, and radiographs on which at least one of the lesser trochanters or teardrop was difficult to define), (2) the surgery was operated with other surgery approaches, and (3) the operative hip with a history of hip surgery or infection.

### Surgical procedure

In DAA and PA surgery, a standard radiographic template was performed using the Orthoview software (Version 6.6.1, Materialise, Leuven, Belgium) to determine component sizing and positioning, level of the neck cut, and amount of leg lengthening or shortening needed was done for patients scheduled for THA.

A tapered, cementless stem and cementless acetabular cups were used in all cases. The Accolade II femoral stem (Stryker, Mahwah, New Jersey, USA), Trident acetabular cups (Stryker), and Pinnacle acetabular cups (DePuy Warsaw, IN, USA) were used for all patients. The surgeon in this study had more than 2000 THAs experience (more than 200 RPA THAs, 500 DAA THAs, and 1000 PA THAs) and performed more than 300 hip replacements annually. The surgeon passed the THA learning curve of the three kinds of operations. The doctor does not have any preference for any procedure. Patients choose which operation to perform freely according to their conditions and costs. But the patients were excluded as candidates for DAA if their body mass index (BMI) was ≥ 30 kg/m^2^.

In our institution, RPA did not add to the patient's cost, so patients are free to choose whether or not to use robot-assisted in their surgery. All the benefits and risks of performing RPA are informed preoperatively to the patients to decide which surgical procedure to perform. The Mako robotic arm interactive orthopedic system (Stryker) assisted surgeons in performing RPA during surgery. Computed tomography (CT)‐based navigation software could directly measure changes in LLD [17].

All RPA surgeries were performed through a posterolateral approach under general anesthesia. After attaching the pelvic arrays, the surgeon began the skin incision and initial exposure. Before hip dislocation, the proximal and a dismal femoral checkpoint were captured to measure the preoperative leg length and hip offset. The surgeon then dislocated the joint and performed the femoral neck osteotomy. The position of the pelvis was confirmed by registering and verifying the position of patient-specific anatomical landmarks displayed on the screen.

The direct anterior approach was performed with the patient in the supine position on a standard operating table. An oblique skin incision starting 3 cm distally and laterally to the anterior superior iliac spine (ASIS) was used. The subcutaneous tissue and the fascia centrally over the tensor fascia lata muscle were divided, followed by blunt dissection to open the interval between the tensor facia lata and the sartorius muscle. The joint capsule was exposed, and the anterior portion was removed. A double osteotomy of the femoral neck facilitated head removal, followed by traditional preparation of the acetabulum using an offset reamer, and the cup was positioned in place. Next, elevate the femur to allow access to the femoral canal. The leg was then placed in external rotation, adducted under the contralateral leg, and the hip was extended by lowering the foot end of the table approximately 30°. The femoral canal was opened, followed by standard preparation using an offset reamer, and the stem was implanted. Leg lengths are checked by palpation of the medial malleoli.

The PA procedures of exposure and osteotomy were described above. The smallest reamer was used to determine the acetabular bottom, then the larger reamers in turn to prepare the acetabulum. The acetabular cup and femoral stem were implanted manually. The lesser trochanter-prosthetic tip distance was measured and checked during the operation to ensure leg length restoration. What’s more, leg lengths are checked by palpation of the patellar. Hip stability and leg length were tested through the full range of motion of the hip.

Stability testing is done with the components in place. Hip stability is tested in extension—first in the abduction and external rotation and then in adduction and external rotation while palpating the femoral head to ensure no impingement or subluxation. Testing in flexion and rotation follows, looking for any posterior subluxation or dislocation. All groups' goals were to restore leg length under the premise of good stability-there is no impingement, dislocation, or subluxation in any hip movement.

**Fig. 2:**
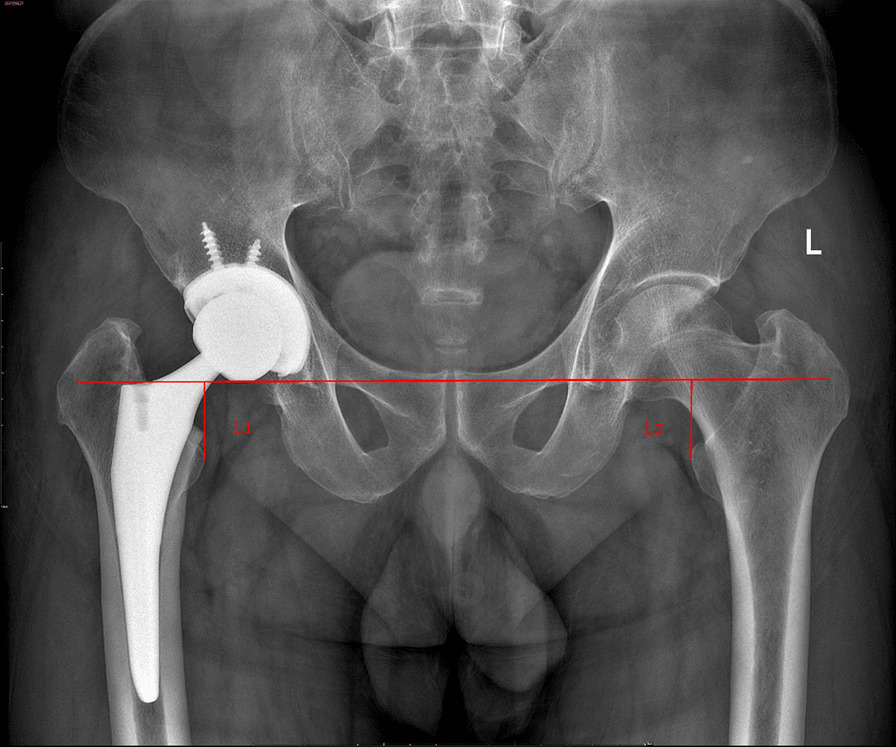
An AP radiograph shows the radiographic measurements of LLD in a male patient. The inter‐teardrop line was used as a pelvic reference. The two lines were drawn, each perpendicular to the teardrop line, starting from the most prominent portion of the lesser trochanter. LLD was defined as the difference in measurement between the operated and reference hip (L1–L2)

All charts and radiographs were retrospectively reviewed to collect information including age, sex, Operation side, height, weight, preoperative Harris hip scores (HHS), and body mass index (BMI). Preoperative and postoperative LLD were measured in the pelvis AP radiographs. Postoperative HHS of patients followed up at two years after surgery (Fig. 2).

### Radiograph measurement

The plain pelvis AP radiographs used in this study were taken in the operating room under anesthesia after surgery, positioning the patient's patella forward.

The radiographic measurements were performed on digital radiographs using the measurement software package by Orthoview software (Version 6.6.1, Materialise, Leuven, Belgium). The contralateral hip was considered as a reference for measurement. Radiographs were calibrated using the known size of each ceramic head as a marker.

The trochanteric technique, as described by Dorr et al. [18], was used to measure the LLD on the low AP pelvis XR. LLD was measured using an inter‐teardrop line as a pelvic reference. The teardrop line was marked bilaterally, creating a horizontal inter-teardrop line across the image. After that, two lines were drawn, each perpendicular to the teardrop line, starting from the most prominent portion of the lesser trochanter. LLD was defined as the difference in measurement between the operated and non‐operated hip. The LLD was given a positive value if the operative limb was longer than the nonoperative limb. Otherwise, the LLD was given a negative value. In patients undergoing bilateral surgery, the first operated lateral was used as a baseline. When calculating the mean value, the direction of length change was not considered (leg lengthened or shortened). To eliminate bias and improve the accuracy of measurement, all the postoperative imaging measurements were done independently by two blinded observers who collected LLD data twice, two weeks apart. The observers were blinded to each other’s results and the type of surgery performed. Each patient’s four measurements were averaged into a single number for LLD, and the absolute LLD values were used in all statistical analyses. There were strong interobserver and intraobserver correlations for all LLD measurements (*r* > 0.82 and *p* < 0.001).

### Statistical analysis

Given the differences in the baseline characteristics between eligible participants in the three groups, propensity score matching (PSM) was used to identify a cohort of patients with similar characteristics. Patients in the three groups were matched with two other groups, respectively. All analyses were performed using IBM SPSS Statistics software (Version 25; IBM, Armonk, New York, USA). The significance level was set at < 0.05 for all tests. Values are expressed as mean ± standard deviation.

When calculating the propensity score by multivariate logistic regression analysis for each patient, we included confounders of age at the time of surgery, sex, body mass index (BMI), and preoperative LLD.

In the matched cohort, paired comparisons were performed using McNemar’s test for binary variables and a paired Student’s t test or paired-sample test for continuous variables. All reported P values are two-sided and have not been adjusted for multiple testing. A post hoc power analysis was performed to compare LLD.

## Results

**Fig. 3:**
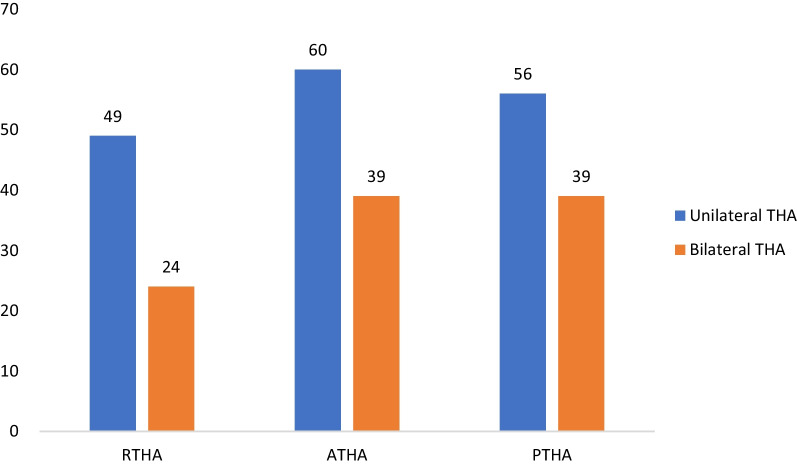
Different number of patients underwent bilateral THA in different groups

We performed analyses on a total of 267 ONFH patients treated with RPA, DAA, or PA.73 patients (27.3%) underwent RTHA with PA, 99 patients (37.1%) underwent DAA, and 95 patients (35.6%) underwent manual PA. In the RPA group, 24 patients underwent bilateral THA (32.9%). In the DAA group, 39 patients underwent bilateral THA (39.4%). In the PA group, 39 patients underwent bilateral THA (41.1%) (Fig. 3).

Using of propensity score matching, 73 patients who underwent RPA were matched with 99 patients who underwent DAA and 95 patients who underwent manual PA, respectively. After that, 95 patients with PA were matched with 99 patients with DAA.

Firstly, propensity score matching was performed between 73 RPA and 99 DAA patients. Based on the propensity score, we generated 1:1 matched cohorts to facilitate comparison between RPA and DAA patients. We matched the patients using the nearest neighbor technique, with a predefined caliper width equal to 0.05 of the standard deviation of the logit of the propensity score, after propensity score matching. A total of 40 patients were included for the propensity score-matched analysis in each group. In the RPA cohort matched with the DAA cohort, 13 patients underwent bilateral THA. In the DAA cohort matched with the RPA cohort, 14 patients underwent bilateral THA (*p* = 0.814). In the first pair of matched cohorts. Mean LLD was 4.10 ± 3.50 mm versus 4.60 ± 4.14 mm in the RTHA cohort compared with matched DAA cohort. There was no significant difference in postoperative LLD between the two cohorts (*p* = 0.577). The power (0.99) of the comparison of different LLD is convincing (Table 1).

**Table 1:** Demographics, postoperative LLD between RTHA and ATHA matched cohorts

Demographics	Robotic THA Mean ± SD (range)	DAA THA Mean ± SD (range)	*p* value
Age (years)	50.21 ± 10.89 (31–69)	50.26 ± 9.35 (27–69)	0.966
BMI (kg/m^2^)	24.41 ± 2.53 (17.63–30.47)	24.62 ± 3.40 (16.90–30.48)	0.737
*Sex*			0.494
Male	23	26	
Female	17	14	
*Side*			0.814
Unilateral	27	26	
Bilateral	13	14	
Peroperative LLD (mm)	− 0.68 ± 1.42	− 0.60 ± 2.17	0.846
Peroperative HHS	55.98 ± 11.41	48.29 ± 19.81	0.081
Postoperative LLD (mm)	4.10 ± 3.50	4.60 ± 4.14	0.577
Postoperative HHS	87.04 ± 7.06	85.33 ± 8.34	0.202

Secondly, propensity score matching was performed between 73 RPA patients and 95 manual PA patients. We also generated 1:1 matched cohorts to compare RPA and manual PA patients. Matching the patients use the nearest neighbor technique, with a predefined caliper width equal to 0.05 of the standard deviation of the logit of the propensity score, after propensity score matching. A total of 58 patients were included for the propensity score-matched analysis in each cohort. In the RPA cohort matched with the PA cohort, 20 patients underwent bilateral THA. In the PA cohort matched with the RPA cohort, 23 patients underwent bilateral THA (*p* = 0.566). In the second pair of matched cohorts. The mean LLD was 3.98 ± 3.27 mm versus 5.38 ± 3.68 mm in the RPA cohort compared with the matched manual PA cohort. There were significant differences in postoperative LLD between the two cohorts (*p* = 0.031). The power (1.00) of comparison of different LLD is convincing (Table 2).

**Table 2:** Demographics, postoperative LLD between RTHA and PTHA matched group

Demographics	Robotic THA Mean ± SD (range)	Manual PA THA Mean ± SD (range)	*p* value
Age (years)	51.15 ± 10. 96 (31–69)	51.88 ± 8.90 (29–72)	0.324
BMI (kg/m^2^)	24.94 ± 3.81 (17.63–31.11)	24.94 ± 3.35 (18.59–36.73)	0.998
*Sex*			0.850
Male	35	36	
Female	23	22	
*Side*			0.566
Unilateral	38	35	
Bilateral	20	23	
Peroperative LLD (mm)	− 0.84 ± 1.424	− 1.00 ± 1.38	0.573
Peroperative HHS	51.56 ± 13.99	49.71 ± 21.80	0.708
Postoperative LLD (mm)	3.98 ± 3.27	5.38 ± 3.68	0.031
Postoperative HHS	89.38 ± 6.81	85.33 ± 8.81	0.019

Thirdly, propensity score matching was performed between 99 DAA patients and 95 manual PA patients. We also generated 1:1 matched cohorts to facilitate comparison between DAA and PA patients. Matching the patients use the nearest neighbor technique, with a predefined caliper width equal to 0.05 of the standard deviation of the logit of the propensity score, after propensity score matching. Seventy-five patients were included in the propensity score-matched analysis in each cohort. In the DAA cohort that matched the PA cohort, 25 patients underwent bilateral THA. In the PA cohort matched the DAA cohort, 24 patients underwent bilateral THA (*p* = 0.862). In the third pair of matched cohorts. Mean LLD was 4.03 ± 3.93 mm versus 5.39 ± 3.83 mm in the DAA cohort compared with matched PA cohort. There were significant differences in postoperative LLD between the two cohorts (*p* = 0.031). The power (1.00) of comparison of different LLD is convincing (Table 3).

**Table 3:** Demographics, postoperative LLD between DAA and PTHA matched group

Demographics	DAA THA Mean ± SD (range)	Manual PA THA Mean ± SD (range)	*p* value
Age (years)	41.77 ± 11.83 (23–69)	42.97 ± 11.47 (20–67)	0.449
BMI (kg/m^2^)	23.45 ± 2.58 (17.90–30.48)	23.90 ± 2.95 (17.30–32.70)	0.152
*Sex*			0.402
Male	44	49	
Female	31	26	
*Side*			0.862
Unilateral	50	51	
Bilateral	25	24	
Peroperative LLD (mm)	− 0.65 ± 1.97	− 0.91 ± 1.43	0.345
Peroperative HHS	59.98 ± 16.55	52.65 ± 19.02	0.126
Postoperative LLD (mm)	4.03 ± 3.93	5.39 ± 3.83	0.031
Postoperative HHS	89.71 ± 6.18	86.91 ± 7.20	0.012

When LLD of more than 3 mm was set as an outlier, 18 RPA, and 18 DAA outliers were in the first pair of matched cohorts (*p* = 1) 0.29 RPA outliers, 37 PA outliers were in the second pair of matched cohorts (*p* = 0.135), 34 DAA outliers, and 48 PA outliers were in the third pair of matched cohorts (*p* = 0.022).

When LLD of more than 5 mm was set as an outlier, 11 RPA, and 10 DAA outliers were in the first pair of matched cohorts (*p* = 0.801) 0.16 RPA outliers, 25 PA outliers were in the second pair of matched cohorts (*p* = 0.024), 21 DAA outliers, and 30 PA outliers were in the third pair of matched cohorts (*p* = 0.122).

When LLD of more than 10 mm was set as an outlier, 4 RPA and 3 DAA outliers were in the first pair of matched cohorts (*p* = 0.694) 0.3 RPA outliers, 6 PA outliers were in the second pair of matched cohorts (*p* = 0.292), 4 DAA outliers, and 7 PA outliers were in the third pair of matched cohorts (*p* = 0.349) (Table 4).

**Table 4:** Different outlier in different matching cohorts

	RPA cohort	DAA cohort	*p* value	RPA cohort	PA cohort	*p* value	DAA cohort	PA cohort	*p* value
3 mm as an outlier	18	18	1	29	37	0.135	34	48	0.022
5 mm as an outlier	11	10	0.801	16	25	0.024	21	30	0.122
10 mm as an outlier	4	3	0.694	3	6	0.292	4	7	0.349

## Discussion

While THA is widely viewed as one of modern medicine's most successful surgical procedures, it is not perfect [5, 19, 20]. LLD after THA remains a significant problem. Our study results showed that RPA and DAA THA were equally effective in minimizing LLD. There was no significant difference in LLD between matched RPA cohort and matched DAA cohort. The LLD in matched RPA cohort and DAA cohort were shorter than matched manual PA cohort. The differences were statistically significant in ONFH patients. Postoperative HHS was also significantly higher in the DAA and RPA cohorts than in the PA cohort.

Early studies suggested that robotic systems have high accuracy [17], which may lead to reduced leg length discrepancies and restoration of the hip centers of rotations and offsets. These reductions in radiographic outliers will likely lead to better clinical outcomes and patient-reported functional outcomes, more durable implant survivorship, and lower rates of complications.

Most studies believed a discrepancy of less than 10 mm did not produce symptoms and was well tolerated [19]. Published studies used various methods to control limb length during THA, including DAA with fluoroscopic guidance; preoperative 2D or, more recently, 3D planning; and robot-assisted intraoperative navigation. To date, only a few studies have been conducted to compare postoperative LLD in RPA, DAA, and PA patients [14]. Bitar et al. study reviewed 67 RPA, 29 DAA, and 59 PA patients with the diagnosis of hip osteoarthritis and showed that all groups achieved a clinically acceptable mean LLD. Their study concluded that the accuracy resulted from the high surgical volume, precise preoperative templating, and intraoperative clinical assessment. However, there were significant differences among different groups of patients at baseline in their study, and their study lacked matching. Unlike their study, our study used a propensity score-matched cohort to compare other groups. Our study increases comparability between groups.

Kayani et al. [15] compared 25 RPA and 50 manual PA performed by one surgeon. Their study shows no difference between robotic-arm-assisted and conventional manual THA when achieving the planned leg length correction. But the sample size of their study was small.

A reason for the accuracy of the DAA group could be attributed to allowing intuitive feedback to the surgeon [21]. Placing the patient in the supine position provides for the combination of both radiographical and palpation checks of the leg length, potentially leading to more accurate leg length. However, this approach has a high learning curve [10, 22, 23]. Surgeon experience might have played an essential role in minimizing LLD regardless of the technique and approach used for THA. The expert surgeon in our study is far beyond his learning curves. Unlike the DAA, robot-assisted surgery monitors limb length in real-time during the operation and compares it contralateral, providing more information to the surgeon via a screen. Our surgical goals are to restore limb length while maintaining hip stability. The results of both techniques are reasonable because they meet the clinical ideal. The difference between different groups may be covered. The findings of the study can not be generalizable to surgeons with less experience or those in the early part of the DAA learning curve. Although prior studies have suggested that there may be some benefit to using robot assistance through THA, our study results indicate that equivalent radiographic outcomes are achievable without using robot assistance. Our study shows that the improved accuracy did not translate into significant LLD improvement compared with the DAA surgery.

We noticed that LLD in matched RPA cohort is shorter than matched manual PA cohort. Postoperative functional scores were also better in the RPA group. When LLD of more than 5 mm was set as an outlier, outliers in RPA cohorts were less than PA cohort. There was a statistical difference between the different cohorts. It shows that RPA has advantages in restoring leg length through the same approach.

Experienced surgeons operating for simple primary surgery will have clinically acceptable LLD, no matter which surgery is performed. But this conclusion cannot be further generalized, especially for beginners or complex cases. It is essential to know that these techniques may harbor risk factors such as more intraoperative complication rates, radiation, blood loss, and operation time [24, 25]. In addition, robotic technology is associated with additional expenses, such as set-up and maintenance overheads, beyond the costs of different operating room times.

The main strengths of this study were that this was a single surgeon study assessing radiological parameters and postoperative function. Outcomes were recorded by blinded observers using standardized techniques with high observer agreement on all outcomes. Because the groups were significantly different at baseline, the best way to compare robot-assisted PA with other surgery operations would be to match similar patients in different groups [26, 27].

This study is not without limitations. Firstly, as a retrospective group study, findings may not be as unbiased as those in a randomized study. Some selection bias might have been part of patient selection, especially after the introduction of the robot. All patients in this study were patients with ONFH because ONFH is one of the very common reasons for performing joint replacement surgery in Chinese patients. In order to increase comparability between groups, patients with ONFH were selected for both groups, but this limits the generalizability of the findings. Secondly, we acknowledge that the study lacks a long-term assessment of clinical outcomes; however, this was beyond the scope of the study. Further studies are needed to investigate this procedure's complications, clinical outcomes, and specific indications. Thirdly, although it has been proposed as a gold standard for LLD measurement, our study has not used full lower body X-rays or EOS X-rays. This is one of the weak points of our study. Future studies should focus on using EOS X-rays to measure leg length. Moreover, robot-assisted technology will apply to the DAA approach surgery to explore whether combing the two techniques can bring better results. DAA, Robot-assisted DAA, and Robot-assisted PA surgery should be compared for differences in leg length restoration. Long-term follow-up should also be included in future studies. Based on this study's post hoc analysis results, the sample size of this study or a larger sample size can still be used.

## Conclusion

This study found that LLD in the RPA cohort is shorter than in the manual PA cohort. But there is no significant difference in postoperative LLD between RPA and DAA operations. Therefore, before one can fully advocate for robotic technology, further research is needed to determine whether robotic assistance will translate into a leg length restoration that justifies the increased cost and operation time.
